# Development of a Microfluidic Device for Exosome Isolation in Point-of-Care Settings

**DOI:** 10.3390/s23198292

**Published:** 2023-10-07

**Authors:** Natasha Ramnauth, Elise Neubarth, Amy Makler-Disatham, Mazhar Sher, Steven Soini, Vivian Merk, Waseem Asghar

**Affiliations:** 1 Asghar-Lab—Micro and Nanotechnology in Medicine Lab, Florida Atlantic University, Boca Raton, FL 33431, USAeneubarth2015@fau.edu (E.N.); amakler@fau.edu (A.M.-D.); 2 Department of Biological Sciences, Florida Atlantic University, Boca Raton, FL 33431, USA; 3 Department of Electrical Engineering and Computer Science, Florida Atlantic University, Boca Raton, FL 33431, USA; 4 Department of Agricultural and Biosystems Engineering, South Dakota State University, Brookings, SD 57007, USA; mazhar.sher@sdstate.edu; 5 Department of Chemistry and Biochemistry, Department of Ocean and Mechanical Engineering, Florida Atlantic University, Boca Raton, FL 33431, USAvmerk@fau.edu (V.M.)

**Keywords:** microfluidics, microfluidic devices, exosomes, exosome isolation, point-of-care (POC), size-exclusion, membrane filters, miRNA detection, exosome purity, exosome size, therapeutics

## Abstract

Exosomes have gained recognition in cancer diagnostics and therapeutics. However, most exosome isolation methods are time-consuming, costly, and require bulky equipment, rendering them unsuitable for point-of-care (POC) settings. Microfluidics can be the key to solving these challenges. Here, we present a double filtration microfluidic device that can rapidly isolate exosomes via size-exclusion principles in POC settings. The device can efficiently isolate exosomes from 50–100 µL of plasma within 50 min. The device was compared against an already established exosome isolation method, polyethylene glycol (PEG)-based precipitation. The findings showed that both methods yield comparable exosome sizes and purity; however, exosomes isolated from the device exhibited an earlier miRNA detection compared to exosomes obtained from the PEG-based isolation. A comparative analysis of exosomes collected from membrane filters with 15 nm and 30 nm pore sizes showed a similarity in exosome size and miRNA detection, with significantly increased sample purity. Finally, TEM images were taken to analyze how the developed devices and PEG-based isolation alter exosome morphology and to analyze exosome sizes. This developed microfluidic device is cost-efficient and time-efficient. Thus, it is ideal for use in low-resourced and POC settings to aid in cancer and disease diagnostics and therapeutics.

## 1. Introduction

Extracellular vesicles (EVs) are molecules with a phospholipid bilayer membrane secreted by prokaryotic and eukaryotic cells. These EVs can be separated into three families: exosomes, microvesicles, and apoptotic bodies [1]. Exosomes are the smallest class of extracellular vesicles, ranging from sizes 30–150 nm. Exosomes are typically characterized by their size; however, they can also be distinguished by their surface charge and density [2]. In addition, exosomes contain various bioactive molecules such as DNA, RNA, metabolites, and cell surface proteins and have been observed to instigate biological responses in extracellular environments [3,4,5]. These bioactive molecules can impact neighboring cells by transferring activated receptors, contributing to epigenetic reprogramming, and stimulating target cells via surface-bound ligands [4]. Therefore, the following stimulation can positively or negatively affect the neighboring cells’ behavior, such as promoting or interfering with the transcription of specific genes. Because of this, exosomes can serve as a mediator of intercellular communication and can initiate tissue crosstalk [6]. Due to these discoveries, exosomes have shown the potential to be novel biomarkers for cancer and various diseases.

As exosomes become more clinically relevant, it is necessary to develop a more streamlined method for the purification and isolation of these extracellular vesicles. Exosomes must be isolated to identify their biomarker content from blood samples, free from interfering polymers or molecules. Various exosome isolation methodologies target the exosome’s physical properties, such as size, immunoaffinity, lipid self-assembly, charge, and density, to isolate them [7]. The chosen exosome isolation method can depend on factors such as sample size, the degree of purity, isolation and retrieval efficiency, cost to carry out methodology, the requirement for specialized equipment, and time [8].

However, current exosome isolation methods are expensive, time-consuming, and require facilities that may be unavailable in resource-constrained settings. Current techniques require a reliable electricity supply, large sample volumes, healthcare infrastructure, refrigeration facilities, expensive biological reagents, and highly trained medical professionals to operate, maintain, and analyze results from sophisticated instruments. These factors can limit exosome isolation methods utilized in clinical, primary research, and low-resourced settings. For example, ultracentrifugation (UC)-based isolation is the most frequently used method for exosome isolation and uses a series of increasing centrifugal forces to isolate exosomes. This technique targets the size and density properties of the exosomes. Density ultracentrifugation is one form of UC-based isolation that requires using a density gradient medium such as iodixanol or sucrose to help purify and separate the exosomes via density. Another form of UC-based isolation is differential centrifugation, known as the gold standard for exosome isolation. Through a series of high centrifugal forces and time, these steps help enhance the purity of the collected exosomes. However, the technique is time-consuming, requires large sample volumes, is labor-intensive, and requires specialized equipment. In addition, the g-force generated from ultracentrifugation can decrease the purity and yield of exosomes [9]. The use of commercial kits is now becoming an attractive option to use due to their rapid processing time, requires very little to no labor, obtains a high yield, and preserves the integrity of the exosomes [10]. However, these commercial kits, such as polyethylene glycol (PEG)-based precipitation, are expensive to obtain and require product refrigeration. Despite this, PEG isolation is not the purist method, as PEG can settle out molecules with the same solubility as exosomes. As a result, the overall sample purity is affected due to protein and nucleic acid contamination precipitated with the exosomes [11]. Size-based separation techniques such as ultrafiltration and size exclusion chromatography (SEC) can separate exosomes based on size or molecular weight [12]. Ultrafiltration utilizes membrane filters to collect the exosomes based on their size, making this method less time-consuming and does not necessarily require specialized equipment compared to ultracentrifugation [13]. SEC uses a column separation approach to filter EVs from plasma [8,11]. This rapid procedure provides a high-purity sample and can process a large sample volume. To further purify samples for clinical use, SEC is often used as a secondary purification step. However, this must be done with caution as the major drawback of SEC is the low yield of exosomes [12,13,14]. Immunoaffinity-based techniques employ the capture of the exosomes via their surface marker proteins. A magnetic bead or nanoparticle is coated with an antibody against a specific exosome surface ligand, such as CD9, CD63, or CD81 [8]. Although the high specificity and high purity may sway researchers to this method, magnetic beads and antibodies are expensive and require technical training to properly prepare [14]. The use of exosomes for therapeutics, vaccines, and biomarkers are hindered by a lack of standardized isolation methods that can reliably harvest and purify a heterogenous population [7].

Microfluidic devices present an alternate option to overcome these challenges. Microfluidics can be a great asset to cancer diagnostics, monitoring, and therapeutics due to their high fluidic control, high sensitivity, and low cost to produce [15]. For POC settings, microfluidic devices must follow the principles of ASSURED (Affordable, Sensitive, Specific, User-friendly, Rapid and Robust, Equipment free and Deliverable to end users), an acronym created by the World Health Organization (WHO) [16]. A device that can provide a prompt and rapid diagnostic can warrant quicker treatment plans for patients and even prevent the rise of mortality rates in underdeveloped countries. There is a dire need to determine a novel method for rapid concentration of exosomes suitable for POC settings that also maintains a high yield, purity, and integrity of the exosomes and their contents.

**Figure 1:**
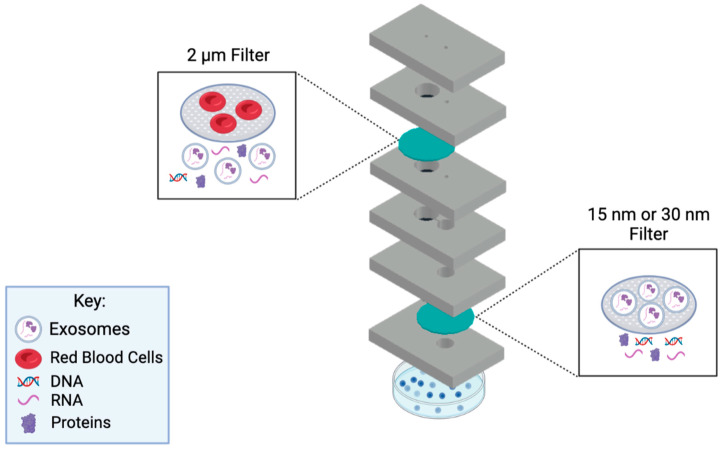
Diagram of the working principle behind the microfluidic device. This novel microfluidic device employs a double filtration approach. A fresh sample will be injected via the sample inlet, which leads to the 2 µm filter for it to be filtrated. Once completed, the sample will pass into the lower chamber and through the channel, to the 15 nm/30 nm filter for further filtration. Exosomes will be collected from the collection channel inlet using a pipette. Schematic created with BioRender.com.

In this work, we present a novel microfluidic device that provides rapid filtration of exosomes from a small volume 50–100 μL, of patient plasma. The assay time is only 50 min, and the device is one-time usable, where it can be conveniently disposed of after use. The main goal is to concentrate exosomes present in small amounts of human biological samples using this developed device, in which the concentrated exosome sample can be used for detection and therapeutic purposes. The device utilizes a filtration method to collect exosomes via size-exclusion principles. Using the filtration methodology allows for a cleaner exosome isolation approach since no reagents and equipment are required to carry out this assay in clinical settings. Hence, the developed microfluidic device employs a double filtration approach to ensure a highly purified post-concentration sample (Figure 1). In this setup, a 2 µm pore-sized filter is used to block the larger biological molecules present in biological samples. Then, a 15 or 30 nm filter is utilized to collect the exosomes. The microfluidic device is cost-efficient, rapid, robust, and user-friendly, making it favorable for POC application. This device could potentially aid in discovering noninvasive diagnostics for cancer and various diseases.

## 2. Materials and Methods

### 2.1. Reagents and Materials

Polymethyl methacrylate (PMMA) with a thickness of 1.5 mm and Double-Sided Adhesive (DSA) were purchased from McMaster–Carr (Atlanta, GA, USA). Whatman Nucleopore Hydrophilic Membrane filters with 2.0 μm pore size, 30 nm pore size, and 15 nm pore size, and with a 25 mm diameter circle each, 1X Phosphate-buffered Saline (PBS), Thermo Fisher’s Total Exosome Isolation Kit (from plasma), Thermo Fisher Total Exosomal RNA, and Protein Extraction Kit were all obtained from Fischer Scientific (Fair lawn, NJ, USA). The Thermo Fisher Taqman miRNA probes were purchased from Thermo Fisher Scientific (Waltham, MA, USA). The 400 mesh Formvar-coated copper grids and the aqueous 2% uranyl acetate were obtained from Electron Microscopy Sciences (Hatfield, PA, USA).

### 2.2. Device Design and Fabrication

**Figure 2:**
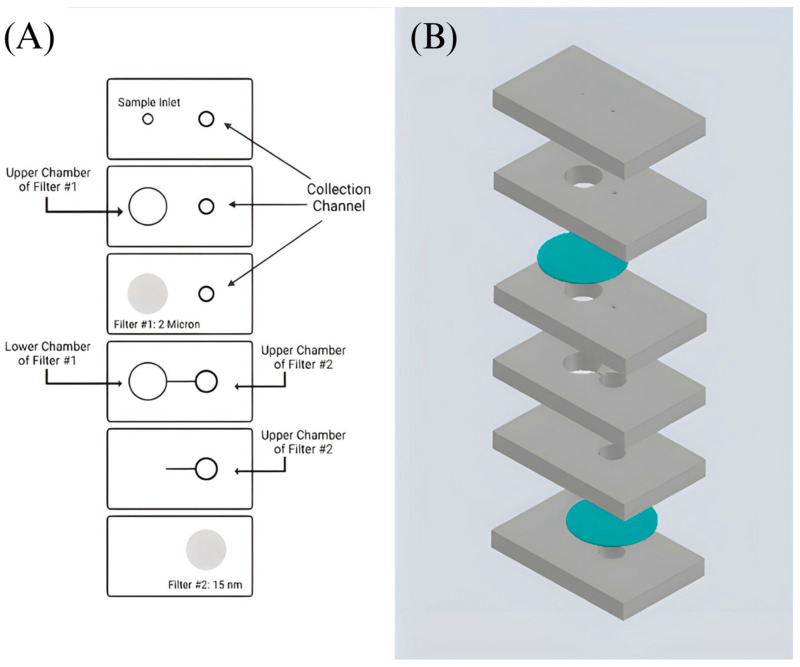
Design of the double filtration microfluidic device. (**A**) Shows a sketch of the developed device’s design. The grey areas indicate the placement of filters #1 and #2. (**B**) Proposed device design in AutoCAD: Version 24.1. PMMA layers are shown in grey, and filters are shown in blue.

**Figure 3:**
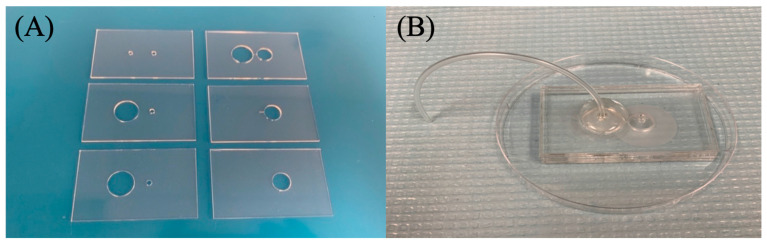
Assembly of double filtration microfluidic device. (**A**) Shows the designed precut PMMA substrates. (**B**) Fully assembled 6-layer PMMA device with filters (2 micron and 30/15 nm pore size) attached. DSA holds each section of the PMMA together.

The device was designed using AutoCAD: Version 24.1 and printed out using a VLS 2.30 CO_2_ laser cutter (Universal Laser Systems, Scottsdale, AZ, USA) that allows cutting of the proposed design from the designated material and ensures reproducibility [17]. The design incorporates a 6-layer precut PMMA substrate with filter #1 sandwiched between the second and third layer and filter #2 sandwiched between the fifth and sixth layer of the device (Figure 2 and Figure 3). It includes an etched channel that leads toward the second filter’s chamber. An etched channel will prevent the backflow of the sample. This device is held together by double-sided adhesive tape. Two inlets are present at the top, with the sample inlet having a diameter of 2.1 mm and the collection inlet having a diameter of 2.5 mm. A Cole-Parmer microbore plastic tubing with an outer diameter of 0.229 m was connected to the sample inlet and sealed using epoxy glue (Figure 3B). Individualized pressing of each layer onto one another with the help of a press machine was also utilized to ensure a leak-proof seal. The device’s dimensions are 65 mm × 40 mm × 9.8 mm. The total assembly time takes approximately 30 min by hand.

### 2.3. Sample Preparation

Deidentified blood samples were obtained from Continental Blood Bank (Continental Services Group Inc., Miami, FL, USA). The samples were spun down at 2000× *g* for 15 min to separate plasma from the blood. For the samples being processed via the device with the 2 µm/30 nm filters, the sample was prepared by diluting 100 µL of patient plasma in 900 µL of 1X PBS. For samples being processed via the device with the 2 µm/15 nm filters, the sample was prepared by diluting 50 µL of patient plasma in 950 µL of 1X PBS. Plasma was stored at −20 °C.

### 2.4. Exosome Isolation Using the Microfluidic Device

The preconcentrated sample was taken up into a 10 mL syringe and injected using a syringe pump. An amount of 1–1.3 mL of 1X PBS was flushed through the device to ensure that any trapped air bubbles, dust, and contamination from the assembly were removed before injection of the sample. Three pieces of DSA were placed on top of the collection inlet to seal the inlet temporarily. An amount of 1 mL of the diluted preconcentrated sample was passed through the device at a 30 µL/min flow rate. An additional 500 µL of 1X PBS was flushed to ensure the whole volume of the preconcentrated sample was passed through. Once completed, the three pieces of DSA were removed, and the concentrated exosomes were collected via a 200 µL pipette connected to the collection inlet on top of the second filter. An additional 200 µL of 1X PBS was resuspended from the second filter to obtain any exosomes that were captured by the filter. The post-concentrated product was then collected for downstream analysis.

### 2.5. Exosome Isolation Using Polyethylene Glycol (PEG)-Based Precipitation

The exosome isolation device capabilities were compared against a standard exosome isolation method, PEG-based isolation. An amount of 100 µL of patient plasma was isolated using the Thermo Fisher Total Exosome Isolation Kit per manufacturer guidelines.

### 2.6. Exosome Size and Purity Characterization

After exosomes were isolated from their respective methods, the samples were subjugated to Dynamic Light Scattering (DLS) analysis for particle size characterization and measuring sample purity. The sample was resuspended repeatedly to prevent aggregation from being picked up by the DLS device and transferred into a cuvette (SARSTED AG & Co., Nümbrecht, Germany). The cuvette was placed into the DLS device (Zetasizer Nano SZ, Malvern, UK), and the parameter was set for its equilibrium process. The set parameter values for the equilibrium process were Temperature: 25.0 °C and Equilibration Time (seconds): 120. Once the equilibrium process was completed, the DLS device was programmed to measure the samples. The measurement parameter values were fixed as follows: Measurement Angle: 90°, Measurement Duration: Manual, Number of Runs: 10, Run Duration (seconds): 10, Number of Measurements: 3, Delay between Measurements (seconds): Zero, and Temperature: 25.0 °C. The size was determined by analyzing the diameter reported by the Intensity PDS (M) percentage of the first peak. In some instances, the 1st peak gave numerical values between 300–2000 nm, indicating the presence of aggregation. Hence, the 2nd peak was also used to determine exosome sizes.

The purity of the sample was analyzed by the PolyDispersity Index (PDI) numerical value. The numerical value ranges from 0.0 (a homogenous sample) to 1.0 (a heterogenous sample with multiple size ranges) [18]. Values < 0.2 are considered to be an accepted PDI value for a sample to be considered monodisperse, while values > 0.7 are heavily broad in size distribution and polydispersal [18,19]. Each condition (exosome concentration from 30 nm device, 15 nm device, and PEG isolation) was repeated three times for precise results. A student’s *t*-test was conducted to observe the significance between exosome size and sample purity across all three conditions.

### 2.7. Transmission Electron Microscope (TEM) Imaging

The samples were vortexed for 30 s prior to TEM sample preparation to prevent aggregation. An amount of 7 µL of the respective exosome sample was placed onto a 400 mesh Formvar-coated copper grid (Electron Microscopy Sciences, Hatfield, PA, USA) and incubated for 5 min at room temperature. Excess exosome solution was removed with a Kimwipe^®^. The grid was then floated on DI water for 1 minute at room temperature to wash off the remaining buffer. Excess fluid was whisked off with a Kimwipe^®^, and then samples were negatively stained with 10 μL of aqueous 2% uranyl acetate for 1 minute before the excess stain was again removed with a Kimwipe^®^. Samples were imaged using a JEOL JEM-1400 series 120 kV Transmission Electron Microscope with an AMT-NanoSprint15L-MarkII camera.

### 2.8. RNA Extraction, cDNA Synthesis, and Real Time-qPCR

Exosomes captured from the microfluidic devices and PEG-based isolation were validated by analyzing exosomal content for the presence of previously established miRNAs [14,20]. Total RNA was extracted from the exosomes utilizing the Thermo Fisher Total Exosomal RNA and Protein Extraction Kit. The purified miRNA was amplified via RTqPCR using the AriaMX PCR system and the Thermo Fisher Taqman miRNA probes include: hsa-miR-93-5p (Assay ID: 478210_mir), hsa-miR-93-3p (Assay ID: 478209_mir), hsa-miR-425-5p (Assay ID: 478094_mir), hsa-miR-425-3p (Assay ID: 478093_mir), and the Taqman Fast Advanced cDNA Synthesis Kit. Additionally, to monitor extraction efficiency, TaqMan miRNA probes cel-mir-2-3p (Assay ID: 478291_mir) and hsa-mir-16-5p (Assay ID: 477860_mir) were used for both exogenous and endogenous controls, respectively.

All three isolation methods were subjected to RTqPCR analysis for their miRNA expression three times to ensure the reliability of results. The miRNA detection is determined by the measurement of the Cq values, which represents a specific miRNA reaching a detectable threshold. Cq values > 35 display an unreliably low detection in RTqPCR for miRNA expression; therefore, those numbers were replaced with a low value of 36 for data analysis [21]. The absence of a Cq value across all three trials was replaced with a 0 to show an undetectable miRNA expression. A student’s *t*-test was conducted to confirm the significance across isolation conditions per each miRNA tested.

## 3. Results

### 3.1. Working Principle of the Double Filtration Microfluidic Device

The first step was to develop a design for the device to provide a double filtration approach for an efficient exosome collection. The device focuses on the principles of filtrating exosomes based on size exclusion, ranging from sizes 30–150 nm. A detailed design of the device and its filter placements are shown in Figure 1. The device’s first filter prevents particles larger than 2 µm from passing to the second filter. The second filter consists of a 15 nm or 30 nm pore size filter membrane in which particles greater than 15 nm or 30 nm are expected to be blocked, while molecules smaller than the filter pore size flow out of the waste chamber into a Petri dish.

The initial device used a 15 nm membrane filter as the second filter to collect the exosomes and filter out any waste. An investigation was conducted to see if using a 30 nm filter for the second filtration achieved a similar or higher exosome yield, purity, and miRNA expressions compared to the 15 nm filter. Hence, filter #2 was interchanged with a 30 nm filter to fulfill these requirements. Particles larger than 30 nm were collected, while particles smaller than 30 nm were discarded as waste. Once the filtration was completed, the exosomes were isolated from the chamber on the top of filter #2, which a pipette was used for exosome collection.

### 3.2. Testing the Device’s Working and Filtering Capabilities

We tested the working capabilities of the device, its exosome isolation abilities, and its ability to process small volumes of blood (50–100 µL). Various increasing flow rates (10, 30, 50, 80, and 100 µL per minute) were tested to see which flow rate was best suitable for passing the plasma samples through both membrane filters. Testing these various flow rates allowed us to determine a rate that met the needs of the 2 µm and 15 nm filter and when it was interchanged with the 30 nm filter. This also allowed us to determine whether the chosen flow rate maintained a stable operating pressure that did not compromise the integrity of the filters, cause any leakage/backflow, or damage the exosomes themselves.

A flow rate of 10 and 30 µL per minute was the most optimal flow rate for the 15 nm device. Visual inspection showed that 1 mL of the diluted sample could adequately pass through the 2 µm and 15 nm filters without rupture or leakage. This observation was also seen in the 30 nm device. A flow rate of 30 µL per minute was the chosen flow rate to concentrate the exosomes as it allowed for rapid processing of the diluted plasma sample without interfering with the integrity of the filters. Table 1 and Table 2 show how the different flow rates affect processing time and the integrity of the 15 and 30 nm membrane filters, respectively.

### 3.3. Comparison of Exosomes Size and Sample Purity via DLS

**Figure 4:**
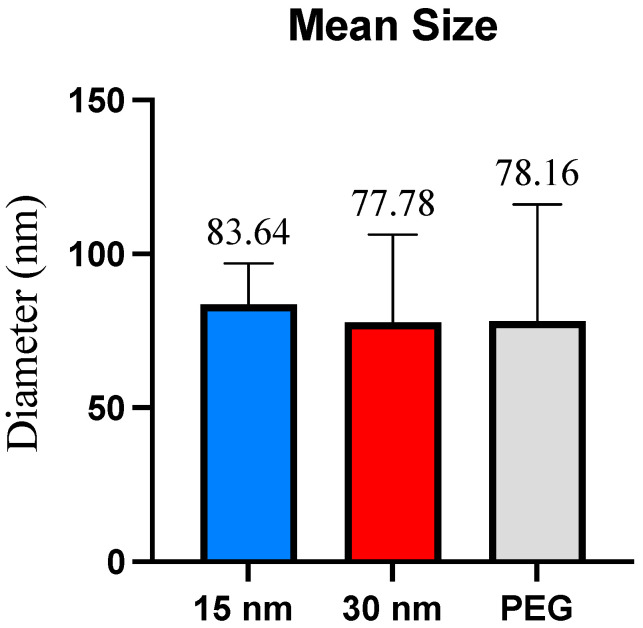
Mean size per exosome isolation method. The mean size represents the diameter (in nm) of the exosomes collected across all three isolation methods. No significant size differences were found.

The exosome size distribution and sample purity were measured using Dynamic Light Scattering (DLS). The first and second highest data peaks were used to determine the average exosome size per condition. Exosomes isolated from the 15 nm filter microfluidic device collected exosomes with an average size of 83.64 nm and showed a size distribution between ~30–100 nm. The 30 nm filter microfluidic device displayed an average size distribution of exosomes between ~40–100 nm, with an average size of 77.78 nm. Exosomes isolated using polyethylene glycol (PEG)-based precipitation, our control isolation method, displayed a size range of ~40–120 nm with an average size of 78.16 nm. A student’s *t*-test revealed no significance in the average size of exosomes collected (Figure 4).

DLS measurements did reveal a size distribution between −150–170 nm. Previous studies have mentioned that exosomes can fall within this size distribution [22,23]. Particles within this range were not factored in the exosome’s averages due to uncertainty about whether these molecules were exosomes, microvesicles, apoptotic bodies, or whether aggregation was present.

**Figure 5:**
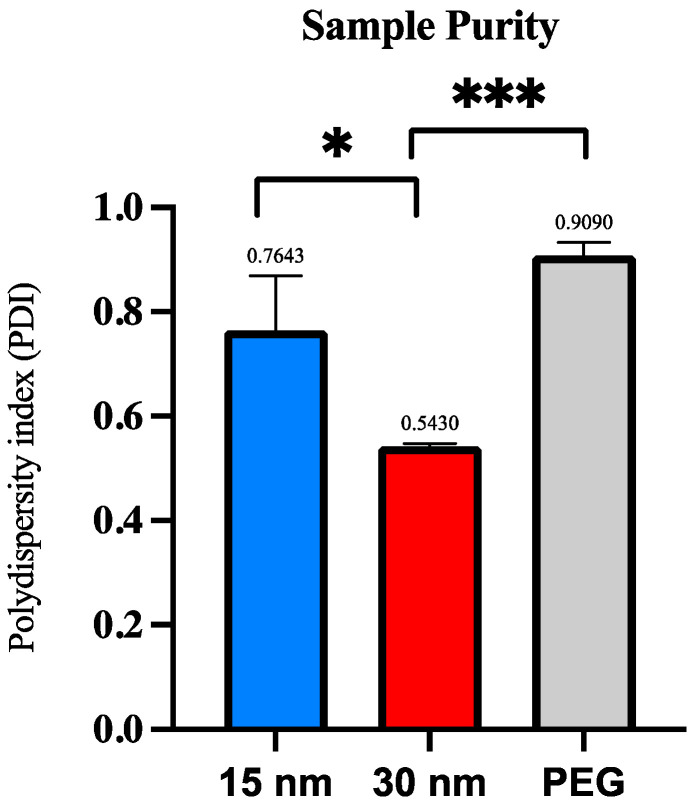
Polydispersity index (PDI) per exosome isolation method via DLS analysis. The polydispersity index (PDI) is compared among the three exosome isolation methods to determine post-concentration sample purity. Significance was determined by conducting a student’s *t*-test. The *p*-value significances are classified as the following: extremely significant (*p* ≤ 0.0001, ***), and significant (*p* ≤ 0.05, *).

The homogeneity of the collected samples was observed by looking at the polydispersity index (PDI). As previously described, the numerical value ranges from 0.0 (a homogenous sample) to 1.0 (a heterogenous sample with multiple size ranges) [17]. Values < 0.2 are considered an accepted PDI value for a sample to be considered monodisperse, while values > 0.7 are heavily broad in size and polydisperse and may not be suitable for DLS analysis [14,17]. Results exhibited that the PDI values for the samples obtained from the 2 µm/15 nm device and 2 µm/30 nm device were 0.764 and 0.543, respectively. When a student’s *t*-test was conducted, the average PDI values for the 2 µm/15 nm device and 2 µm/30 nm sample purity were significant (*p* ≤ 0.05). Plasma samples isolated using PEG showed a PDI value of 0.909. Another *t*-test was conducted for the average PDI values between the 2 µm/30 nm compared to PEG, which showed an extremely significant value (*p* ≤ 0.0001). The 2 µm/15 nm device compared to PEG showed no significance. Figure 5 summarizes the PDI across all three conditions.

### 3.4. Exosomal miR Expression from Microfluidic Device and PEG via RTqPCR

A standard has been previously established in our lab for collecting and analyzing exosome miRNA from patient samples, hence miR-425-5p, miR-425-3p, miR-93-5p, and miR-93-3p, with miR-16-5p serving as the exosomal endogenous control [21]. All conditions will be examined for their expression levels, as this will serve as a vital parameter to assess the recovery rate of the genetic materials per all three isolation conditions.

For miRNA obtained from exosomes extracted from the 15 nm device, miR-16-5p exhibited a relatively higher expression level compared to the other miRNAs tested within this condition, with an average Cq value of 23.93. Subsequently, miR-93-5p exhibited an average Cq value of 28.89, with miR-425-5p and miR-425-3p showing similar expression levels, of Cq values of 31.46 and 31.52, respectively. miR-93-3p did appear much later in the PCR amplification procession, displaying an average Cq value of 35.22. However, as mentioned before, Cq values > 35 is considered an unreliable expression level and should not be considered for diagnostic and therapeutic purposes.

For miRNA assessed from exosomes isolated from the 30 nm device, the control miR-16-5p continued to show upregulation level compared to the other miRNAs tested within this condition, with an average Cq value of 24.99. miR-425-5p and miR-93-5p did also display a similar expression level of 29.94 and 29.61, respectively. The expression level of miR-425-3p was relatively downregulated, displaying an average Cq value of 34.78. In contrast, miR-93-3p was not detectable amongst the three trials, suggesting either the absence of the miRNA or the expression levels fall below the detectable threshold level. More analysis will need to be conducted to further verify this claim.

In the final condition, miRNA from exosomes extracted using a PEG reagent were assessed. Interestingly, the detection of miR-16-5p occurred at a Cq value of 30.93, indicating relatively lower detection compared to the devices. miR-425-5p and miR-93-5p presented similarly downregulated Cq value of 33.49 and 34.34, respectively. In contrast, miR-425-3p was not detectable amongst the three trials, and miR-93-3p exhibited an average Cq value of 35.11, making it an unreliable measurement.

**Figure 6:**
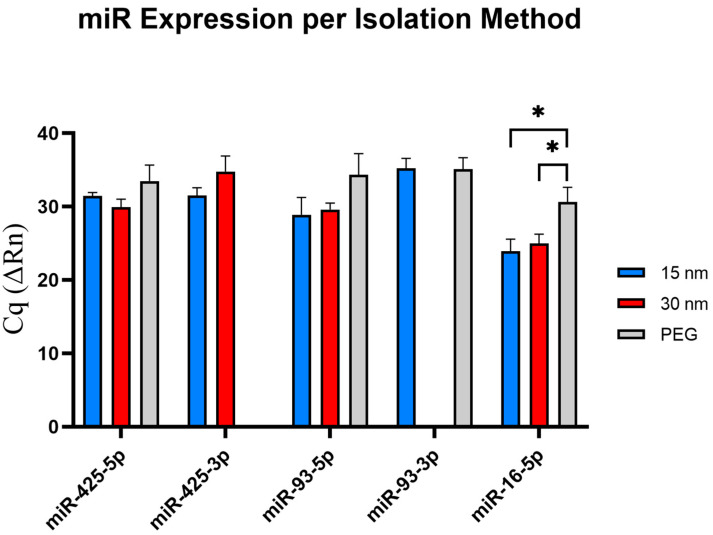
Validation of miRNA expression via RT-qPCR. The selected exosomal miRNA are shown in this figure to investigate how different isolation methods affect miRNA expression. miR-4-3p expression was undetectable from exosomes isolated via PEG isolation, and miR-93-5p expression was undetectable from exosomes isolated via 2 µm/15 nm device. The *p*-value significances are classified as the following: significant (*p* ≤ 0.05, *).

As presented in Figure 6, miR-425-5p was expressed amongst all three conditions with no variation in expression levels. Expression of miR-425-3p from exosomes isolated from both the 15 nm and the 30 nm filter devices was apparent, with miR-425-3p showing slightly earlier from exosomes collected in the 15 nm device than the 30 nm device; however, no Cq detection was observed from exosomes isolated using PEG. No significant differences were found among Cq expression levels between both filter devices only. Notably, miR-93-5p displayed similar Cq values from exosomes isolated from both the 15 nm and 30 nm devices, indicated its early appearance compared to PEG. There were no significant findings across all three isolation conditions. Expression of miR-93-3p from exosomes isolated from the 15 nm filter device and those isolated by PEG showed Cq value expression at ~35 Cq value, and no Cq detection was observed from exosomes isolated by the 30 nm device. With miR-16-5p serving as the exosomal endogenous control, CQ values < 35 were present, indicating that both the 15 nm and 30 nm devices can successfully isolate exosomes from just 50–100 µL of plasma sample alongside PEG. The miR-16-5p expressions were statistically significant (30 nm filter and PEG, *p* ≤ 0.05: 15 nm filter and PEG, *p* ≤ 0.05).

### 3.5. Exosome Morphology and Characterization Using TEM Imaging

**Figure 7:**
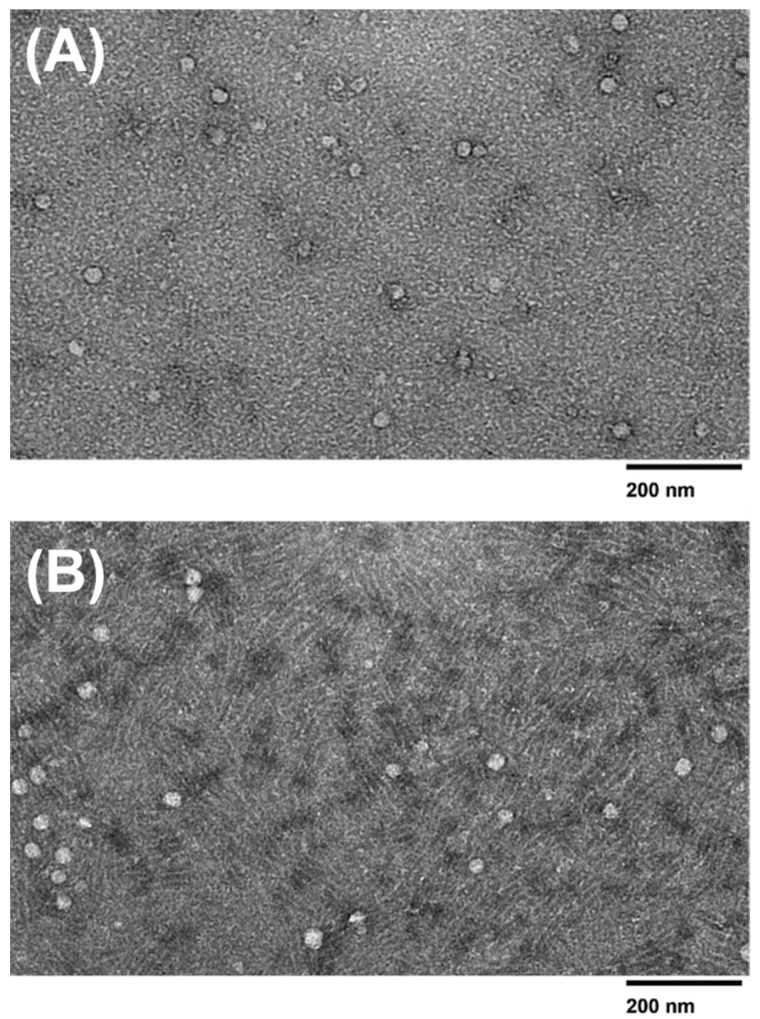

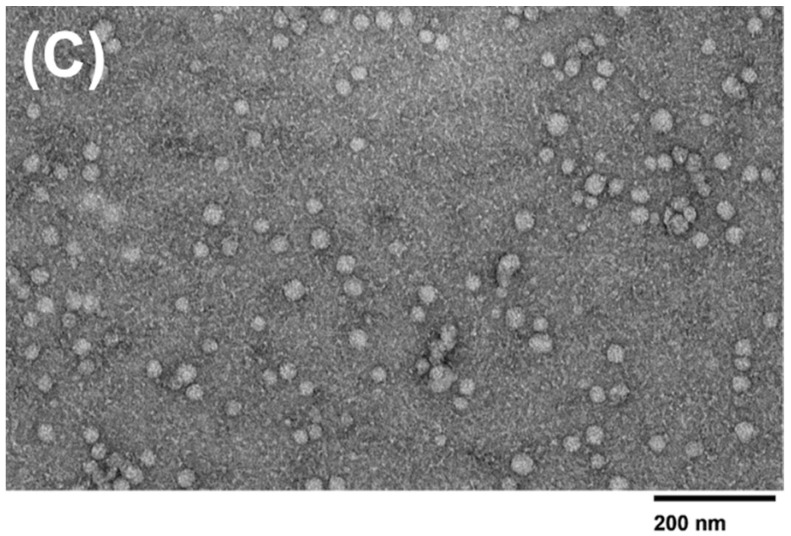
TEM images of isolated exosomes. TEM images (120 kV) of exosomes isolated using the (**A**) 15 nm device, (**B**) 30 nm device, and (**C**) PEG isolation method.

The morphology and size distribution of the microfluidic device and PEG-isolated exosomes were determined using TEM imaging. TEM data show that the extracellular vesicles collected from all three methods displayed a smooth round shape. Exosomes collected from the 2 µm/15 nm device displayed a size distribution of ~30–95 nm in diameter. It was also observed that exosomes collected from the 2 µm/30 nm showed sizes of ~30–55 nm in diameter. The devices compare well with PEG, as the exosomes collected via PEG showed sizes of ~30–100 nm. However, since PEG is a sticky molecule, it increases the chances of aggregation within the sample, making it difficult to distinguish two exosomes from each other. As shown in Figure 7C, the PEG-isolated sample exhibits a significant increase in aggregation compared to the 30 nm and 15 nm device-isolated exosomes (Figure 7A,B). In addition, we qualitatively observed that more exosomes were present in the samples that were isolated with PEG compared to the devices. However, the number of exosomes isolated using either the 30 nm or 15 nm device did not show any significant difference between each other.

## 4. Discussion

Although in its nascent stages, microfluidics can be a great asset to cancer diagnostics, monitoring, and therapeutics due to its high sensitivity, and relatively low cost to produce [15]. Diagnostics and monitoring depend on quantifying proteins and the presence of biomarkers such as circulating tumor cells, proteins, DNA, RNA, and miRNAs [24]. Creating a platform that can rapidly and quickly detect specific biomarkers from small volumes of samples at a low cost can revolutionize the cancer field. Circulating tumor cells (CTCs) that detach from tumors can circulate in the bloodstream, indicate disease, and be used for monitoring [25]. One study has developed a silicon microfluidic device called the “CTC-chip” to detect CTCs from peripheral blood from lung cancer patients. This device could capture rare CTCs from the plasma sample and capture 98% of viable CTCs. This device can be a quick and cheap alternative for diagnostics and monitoring lung cancer patients [26]. Another study has shown the ability of microfluidic immunomarker devices to detect CA15-3, a protein biomarker found in breast cancer patients. This disposable device detected CA15-3 in samples as low as 6.0 µU mL^−1^ from 2 uL of serum sample, making it a promising method for detecting CA15-3 in breast cancer patient samples [27]. Even with their captivating advantages, such devices have yet to be employed in POC settings, where challenges such as commercialization and interactions between the device and sample can lead to inaccurate results [28].

There has been growing evidence that exosomes participate in intracellular communication and can promote metastasis, angiogenesis, and proliferation of cancer. There is also a plethora of research in exosome isolation that aims to combat the present-day challenges of their isolation methods, such as the use of expensive reagents, heavy and bulky equipment, and extensive processing time. However, there is insufficient research that analyzes their robustness and sensitivity for downstream clinical analysis. Additionally, a better standardized process to selecting an exosome isolation method can reduce the amount of inconsistences and uncertainties seen in exosome research [12]. This hinders the exosome’s remarkable potential for incorporation into clinical practices. Moreover, some research projects fail to emphasize how these proposed isolation methods affect the detection of exosomal content for additional clinical relevance. Early detection is the most efficient way to improve diagnostics. Exosomal biomarkers are key to understanding how exosomes promote diseases. By studying their biomarker components, it opens the door to the development of novel, noninvasive diagnostic and therapeutic markers.

This current study presents a novel exosome isolation method utilizing a double filtration microfluidic platform that is appropriate for POC settings, rapidly isolates exosomes, and yields a high purity and recovery of exosomes. In addition, this platform can isolate exosomes well enough to detect exosomal biomarkers for downstream analysis. A double filtration approach was taken to ensure a cleaner process of isolating exosomes without reagents that require refrigeration or are expensive to acquire. Because the exosomes are being filtered via size-exclusion principles, the selected size filters for the double filtration device must meet the desired exosome size characteristics and ensure a pure collection of exosomes. Hence, a 2 µm and a 15 nm filter were utilized to achieve exosome collection. As far as it is known, this device is currently the first reported microfluidic device to use a 15 nm pore size filter for exosome isolation. Exosome isolation microfluidic devices have been reported to use 30 nm pore size filters as the smallest filter incorporated into these devices [29,30]. To fully understand why this may be, a device using a 2 µm and a 30 nm filter was made to compare exosome size, purity, and difference in miRNA expression against the 2 µm and 15 nm filter device.

It was observed that there was a significant difference between the purity levels of the post-concentration exosome sample obtained from a 15 nm pore size filter compared to a 30 nm pore size filter, with the 30 nm device yielding a purer sample. Because the 15 nm pore size is much smaller, it is more prone to filter blockage caused by large polymers and molecules. As exosomes range in various sizes between 30–150 nm, the 15 nm filter could have been clogged rapidly due to its smaller pore size, thus preventing drainage of cellular waste. Microvesicles can range from 100–1000 nm, and apoptotic bodies can range from sizes to 50 nm–5 μm. There is a possibility that the membrane filters could have captured these and affect the purity [1]. The overlap between exosomes and microvesicles can yield an impure sample and decrease the specificity of the exosomes collected in the sample. Due to potential filter clogging issues and the overlap between exosomes and microvesicles, the 30 nm filter-based devices may be a better choice for exosome isolation. According to DLS analysis, there was no difference in exosome size presented between the 15 nm pore size and the 30 nm pore size membrane filters, as both filters yielded sizes ranging varying from ~30–100 nm, with the 15 nm device and the 30 nm device yielding an average size of 83.64 and 77.78, respectively. The observed smaller sizes obtained within the 30 nm filter could be accredited to the depletion of exosome aggregates or larger complexes, which may have been captured within the 15 nm filter. However, it is important to consider other factors, such as the variability in exosome sizes and the accuracy of the filter pore sizes to understand the observed size differences fully. The TEM analysis revealed that no molecules surpassed the size range of microvesicles (~100–1000 nm) and that the 15 nm device, 30 nm device, and PEG-based isolation all displayed various exosome sizes between ~30–100nm. The double filtration device proposed in this study successfully isolated exosomes that fell within the appropriate size ranges.

It is also shown that microfluidic devices can isolate exosomes similar in size to those isolated by the PEG reagent. Although the average exosome size in the 15 nm was slightly higher, the 30 nm device and the PEG-isolated exosomes’ average sizes were similar. The 15 nm device displayed a size range approximately ~30–100 nm in DLS analysis, while displaying a size range of ~30–95 nm from the TEM analysis, while the 30 nm device showed sizes ranging from ~40–100 nm from DLS analysis and ~30–55 nm from TEM analysis. The PEG-based isolation did collect a slightly larger exosome size distribution which fell between ~40–120 nm and TEM analysis showed distribution range ~30–100 nm. The difference in average size across all three isolation methods was not significant. Despite PEG’s size range falling under the classification of exosomes, the vesicles ranging between ~100–150 nm are difficult to determine whether these are exosomes or microvesicles due to the overlapping of their sizes. Due to PEG being a notoriously sticky molecule, it increases the chances for aggregation, which could potentially account for the larger size distribution displayed from the DLS analysis. The discrepancies between the size ranges obtained from the DLS and TEM are noted. The difference in detection principles per technique is attributed to these size discrepancies, as DLS measures the overall hydrodynamic radius of the particles in the sample, while TEM imaging focuses on the measurements of the individual exosomes. Sample preparation and device sensitivity could also attribute the difference in exosome sizes. There was an extremely significant difference between the size distribution of the post-concentration sample obtained from the 30 nm filtration device and PEG isolation. 

An amount of 100 µL of plasma samples isolated using PEG showed a significantly higher polydispersity index of 0.909 compared to the 30 nm device, showing a PDI of 0.543. No significance was observed between the sample’s polydispersity obtained from the 15 nm device and PEG isolation. PEG isolation focuses on enriching molecules by targeting their solubility properties. This allows molecules with similar solubility properties to exosomes to also precipitate out, leading to an impure sample. Because of PEG’s properties, the exosomes and molecules present in the sample can aggregate with each other, skewing the results for both size and purity.

MiRNA plays a significant role in influencing human health, prompting scientists to study various miRNAs on their impression on cancers and diseases. Therefore, analyzing the miRNA expression patterns per isolation condition can allow us to further develop more effective biomarkers for diagnostics and therapeutics. In this study, we focused on investigating the expression levels of miR-425-5p, miR-425-3p, miR-93-5p, and miR-93-3p. The selection of these miRNAs was driven due to their relevance in cancer biology and as cancer diagnostics and prognostic markers. As reported, miR-425, especially miR-425-5p, has been known to promote the migration and proliferation of various cancers such as ovarian cancer, renal cell carcinoma and prostate cancer, while miR-425-3p has been upregulated in colon cancer patients, which could serve as a prognostic marker and be used as an anti-tumor therapy target [31,32,33,34]. Similarly, miR-93-5p has been shown to promote pro-tumorigenic effects and has been upregulated in cancer cells, such as PDAC, with miR-93-3p also showing similar results, serving as a biomarker for triple negative breast cancer [35,36,37]. By testing these miRNAs, this would help validate the detection of these chosen miRNAs from patient samples. Interestingly, when testing the respective exosomal miRNAs, we found that three out of five exosomal miRNAs tested (miR-425-3p, miR-93-5p, and miR-16-5p) displayed an earlier detection compared to the other isolation methods, while only one miRNA (miR-425-5p) showed an earlier detection in the 30 nm device. Interestingly, no detection of miR-425-3p was found from exosomes isolated via PEG but was detected in exosomes obtained from both 15 nm and 30 nm devices. With miR-93-3p, no detection was found in exosomes isolated from the 30 nm filter device, while expression levels were observed in exosomes isolated from both the 15 nm filter device and PEG isolation. Further investigation will need to be conducted to determine the underlying reasons for these variations. With miR-16-5p serving as the endogenous control, confirming the presence of exosomes collected, and utilizing the expression levels of the miRNA, this allows for the assessment of the recovery rates from all three tested isolation methods. Notably, both pore-size microfluidic devices showed an upregulated miRNA expression compared to PEG. These findings insinuate that the microfluidic devices may offer a higher recovery rate of exosomes present in the sample, demonstrating their potential to be used for downstream analysis.

As previously mentioned, the 15 nm device requires only 50 µL of plasma diluted in PBS, for optimal operation, compared to the 100 µL required for the 30 nm device. With the 15 nm filter device displayed an upregulation for three miRNAs (miR-425-3p, miR-93-5p, and miR-16-5p) from 50 µL, suggesting that 15 nm microfluidic device is more sensitive, offers a higher recovery rate of exosomsal miRNAs, and can be effective in the detection of exosomal miRNA compared to both the 30 nm device and PEG.

The developed microfluidic platform contains numerous advantages to its design and fabrication process, making it attractive for mass production. The device focuses on the principles of size exclusion and double filtration to capture the exosomes, a cleaner alternative compared to using expensive reagents or requiring refrigeration equipment. Secondly, the design platform contains a double filtration approach to enhance the purity of the post-concentration sample. This can mitigate the need to combine two different exosome isolation methods to enhance purity. Thirdly, the selection of the PMMA and the hydrophilic membrane filters are cost-efficient, biocompatible, and commercially available for mass production facilities. Also, the hydrophilic membrane’s rigidity and durability do help mitigate filter rupture and is considered a well-established filter to be used for exosome isolation methods, allowing for the basis of comparison and reproducibility across different studies. In addition, the design can easily be reproduced. Finally, the device’s usability is significantly easier to use than its costly and bulkier alternatives, as the only equipment required to operate the device is a syringe pump.

The microfluidic device’s primary aim is to be applied in POC settings for downstream processing and to see if it can present itself as a superior isolation method compared to PEG-based isolation. The microfluidic device can rapidly process small quantities of samples, making it optimal for POC settings. The device can process fingerpick quantities (10–100 µL) of plasma within one hour. This is a similar timeframe to the commercially available Total Exosome Isolate Kit (from Plasma). However, the kit requires ultracentrifugation, which can deteriorate the exosome’s integrity. The commercial Total Exosome Isolation kit for plasma is expensive, while the microfluidic devices would be inexpensive, supplying a cost-effective isolation method. The microfluidic device is a more user-friendly method than PEG-based isolation. The microfluidic device works optimally when the plasma sample and 1X PBS are at room temperature, eliminating the need for external storage instrumentation. In contrast, the PEG reagent requires refrigeration and centrifugation for long-term storage. The syringe pump is the only instrumentation needed to operate the microfluidic device. A syringe pump offers a user-friendly approach to operation and can accommodate any syringe size found in a medical facility. Without electricity in low-resourced settings, a battery-powered syringe pump could be utilized or manually injected by hand, eliminating the need for any equipment. This developed microfluidic device is sensitive, user-friendly, cost-friendly, rapid, and robust, which meets the principles of ASSURED (Affordable, Sensitive, Specific, User-friendly, Rapid and Robust, Equipment free, and Deliverable to end users) [20].

Additionally, our devices aim to combat some of the most pressing challenges of exosome isolations using filter members, such as filter membrane blockage. We optimized a controlled flow rate and filter pressure using a syringe pump to minimize clogging. A higher flow rate does allow for the filters to get clogged at a quicker rate, which can rupture the membrane itself. Next, we employed a pre-filtration step by passing PBS through the device to ensure that any particles that may have been capture during the building process is removed. This step allows us to prewet the filters to ensure a smooth passing of the diluted sample. Furthermore, we diluted patient plasma within PBS before filtration, using only 50–100 µL of patient sample. By doing so, this allows for a smoother passage through the filters without diminishing its integrity. These combined measures have allowed for an efficient filtration and prevents filter membrane rupture within the device.

The devices discussed in this paper present limitations that future work and investigation can further improve upon. The experiments with these devices were done only with healthy patient plasma. Even though these experiments have been successful, testing the device using cancer samples may pose unforeseen issues. For example, cancerous samples are known to have a higher concentration of molecular content, such as circulating tumor cells and free-floating content. Cancer cells also tend to secrete a higher concentration of exosomes [38]. The 15 nm filter and potentially the 30 nm filter may not have the ability to withstand the higher concentration of exosomes, allowing for more sample impurity and increased chances of filters rupturing during exosome isolation. In addition, diluting the sample can cause low throughput. Due to this, the low concentration may need to undergo additional analysis time or be combined with other purification sources to achieve an adequate signal. It is unknown whether the devices can isolate exosomes from whole blood itself or from other complex samples. To overcome this, future work could involve a design revision that allows the microfluidic device to handle parallel sample processing or increase the capacity of the chambers, allowing to process more sample volume. This approach can considerably increase throughput and yield. Since the device’s purpose is to be used in POC settings, isolating plasma from patient blood requires centrifugation. Centrifuges are found in virtually every medical laboratory, where a low-speed centrifuge that is inexpensive can serve as a replacement. The results presented in this paper were compared against a traditional form of exosome isolation using a PEG reagent. However, it is unknown whether these devices would show similar results when compared to the other exosomal isolation methods, such as ultracentrifugation. Another limitation to this study was the use of DLS equipment instead of Nanoparticle Tracking Analysis (NTA) instrument for measuring the size distribution and concentration of particles. Future research endeavors should focus on finding alternative exosomes quantification methods such as Tunable Resistive Pulse Sensing (TRPS), Vesicle Flow Cytometry, Surface Plasmon Resonance (SPR), or Electron Microscopy (EM) [39]. It would also be beneficial to conduct a comparative analysis to investigate miRNA, proteomic and genetic signatures from EVs isolated from cancer and healthy patient samples and analyze their signatures using the developed microfluidic device against PEG isolation. Future investigations should analyze the effectiveness of the device’s ability to isolate other nanoparticles such as very low density lipoprotein (VLDL) particles and microvesicles as the sizes overlap with exosomes, influencing downstream analysis. 

## 5. Conclusions

In summary, exosomes offer a remarkable avenue to develop novel diagnostics for an array of illnesses. Research must focus on developing a consistent and reliable method for extracting exosomes from biofluids. In this paper, we have developed a double filtration microfluidic device that can isolate exosomes within 50 min from a small volume of 50–100 µL of plasma. The selection of a 15 nm or 30 nm size pore membrane depends on if we are looking for a sample that yields a higher detection in miRNA or yields a purer sample. Regardless, PEG isolation is costly compared to the microfluidic device, which is cost-efficient, time-efficient, and user-friendly, making it suitable for POC settings to aid in downstream applications.

## Figures and Tables

**Table 1:** Optimization of flow rate for 2 micron/15 nm device: The flow rate selection is determined by observing if 50 µL of plasma diluted in 950 µL of 1X PBS can adequately pass through both filters during the sample filtration and post-washing step. Because time is a critical factor for a diagnosis, determining a rate that is not time-consuming is essential. Hence, a flow rate of 30 µL per minute was selected.

Flow Rate	Time (Sample Filtration + Post Washing Step)	Observation
10 µL/min	2.5 h	Sample passed through both filters; drainage was observed
30 µL/min	50 min	Sample passed through both filters; drainage was observed
50 µL/min	30 min	Filter ruptured during sample filtration
80 µL/min	20 min	Filter ruptured during sample filtration
100 µL/min	15 min	Filter ruptured during sample filtration

**Table 2:** Optimization of flow rate for 2 µm/30 nm device: As with the 15 nm device, the selection of the flow rate must be optimized to where it would allow 100 µL of diluted plasma in 900 µL of 1X PBS to flow through both the two µm filter and the 30 nm filter. The 30 µL/min flow rate was also chosen for the 30 nm device as this met the demands for allowing the filtration through both filters in a time-friendly filtration process.

Flow Rate	Time (Sample Filtration + Post Washing Step)	Observation
10 µL/min	2.5 h	Sample passed through both filters; drainage was observed
30 µL/min	50 min	Sample passed through both filters; drainage was observed
50 µL/min	30 min	Filter ruptured during post-washing step
80 µL/min	20 min	Filter ruptured during post-washing step
100 µL/min	15 min	Filter ruptured during sample filtration

## Data Availability

Not applicable.
